# Empiric Potassium Supplementation and Increased Survival in Users of Loop Diuretics

**DOI:** 10.1371/journal.pone.0102279

**Published:** 2014-07-16

**Authors:** Charles E. Leonard, Hanieh Razzaghi, Cristin P. Freeman, Jason A. Roy, Craig W. Newcomb, Sean Hennessy

**Affiliations:** 1 Center for Clinical Epidemiology & Biostatistics, Department of Biostatistics & Epidemiology, Perelman School of Medicine at the University of Pennsylvania, Philadelphia, Pennsylvania, United States of America; 2 Center for Pharmacoepidemiology Research and Training, Perelman School of Medicine at the University of Pennsylvania, Philadelphia, Pennsylvania, United States of America; 3 Department of Pharmacology, Perelman School of Medicine at the University of Pennsylvania, Philadelphia, Pennsylvania, United States of America; University of Florida, United States of America

## Abstract

**Background:**

The effectiveness of the clinical strategy of empiric potassium supplementation in reducing the frequency of adverse clinical outcomes in patients receiving loop diuretics is unknown. We sought to examine the association between empiric potassium supplementation and 1) all-cause death and 2) outpatient-originating sudden cardiac death (SD) and ventricular arrhythmia (VA) among new starters of loop diuretics, stratified on initial loop diuretic dose.

**Methods:**

We conducted a one-to-one propensity score-matched cohort study using 1999–2007 US Medicaid claims from five states. Empiric potassium supplementation was defined as a potassium prescription on the day of or the day after the initial loop diuretic prescription. Death, the primary outcome, was ascertained from the Social Security Administration Death Master File; SD/VA, the secondary outcome, from incident, first-listed emergency department or principal inpatient SD/VA discharge diagnoses (positive predictive value = 85%).

**Results:**

We identified 654,060 persons who met eligibility criteria and initiated therapy with a loop diuretic, 27% of whom received empiric potassium supplementation (N = 179,436) and 73% of whom did not (N = 474,624). The matched hazard ratio for empiric potassium supplementation was 0.93 (95% confidence interval, 0.89–0.98, p = 0.003) for all-cause death. Stratifying on initial furosemide dose, hazard ratios for empiric potassium supplementation with furosemide <40 and ≥40 milligrams/day were 0.93 (0.86–1.00, p = 0.050) and 0.84 (0.79–0.89, p<0.0001). The matched hazard ratio for empiric potassium supplementation was 1.02 (0.83–1.24, p = 0.879) for SD/VA.

**Conclusions:**

Empiric potassium supplementation upon initiation of a loop diuretic appears to be associated with improved survival, with a greater apparent benefit seen with higher diuretic dose. If confirmed, these findings support the use of empiric potassium supplementation upon initiation of a loop diuretic.

## Introduction

Furosemide was introduced in the 1960s and is very widely used to treat heart failure and edema [1], [2]. Furosemide and other loop diuretics cause urinary potassium loss [3], which can lead to potassium depletion and might be expected to increase mortality by mechanisms including ventricular ectopy [4], [5]. On this basis, among others, the 2000 National Council on Potassium in Clinical Practice recommended that potassium supplementation be routinely considered in persons with hypertension receiving a non-potassium sparing diuretic, and in persons with heart failure even if normokalemic [6]. However, no studies have examined the efficacy or effectiveness of empiric potassium supplementation (defined as preventive/prophylactic supplementation, in contrast to repletion reactive to serum potassium laboratory results) on reducing the risk of adverse clinical outcomes in users of loop diuretics. As a result of this evidence gap, a 2012 evidence review recommended against the routine use of empiric potassium supplementation in patients receiving loop diuretics [7], despite the aforementioned practice guidelines. A randomized trial addressing this important question seems unlikely. To provide evidence to help inform this common clinical decision, we sought to examine the effectiveness of empiric potassium supplementation in reducing all-cause mortality in a cohort of new users of loop diuretics. Recognizing that the effects of empiric potassium supplementation might vary by diuretic dose [7], we wished to examine the effect stratified on diuretic dose. We secondarily examined the effectiveness of empiric potassium supplementation in reducing a composite endpoint of sudden cardiac death/ventricular arrhythmia (SD/VA) to look for mechanistic evidence.

## Methods

### Ethics statement

This observational study was approved by the University of Pennsylvania's institutional review board.

### Overview and study population

We performed a propensity score-matched cohort study of new users of loop diuretics. Our cohort consisted of person-time exposed to a new course of a loop diuretic (bumetanide, ethacrynic acid, furosemide, or torsemide). Data for this study included that of the Medicaid programs of California, Florida, New York, Ohio, and Pennsylvania from 1999–2007 [8]. These states comprise about 38% of the United States Medicaid population [9], with the nine-year dataset recording the experience of over 48 million cumulative enrollees and 108 million person-years (p-y) of observation. Because up to 27% of Medicaid beneficiaries (varying by state and year) were co-enrolled in Medicare (i.e., dually enrolled) [10]–[12], we also obtained Medicare claims (including Part D data from 2006 onward) to ascertain a more complete picture of enrollees' healthcare. We linked these data to the Social Security Administration Death Master File to ascertain deaths.

### Defining the study cohort: identification of incident loop diuretic courses

We defined new users of loop diuretics as those with at least a 12-month baseline period of Medicaid enrollment before the first loop diuretic prescription was filled. Beneficiaries meeting any of the following criteria were excluded: a) incident loop prescription was a liquid dosage form, as their inability to swallow a solid dosage form may have been indicative of functional impairments that are not reliably captured in administrative data; b) use of another diuretic class during the baseline period except for thiazide or thiazide-related agent use (hereafter simply referred to as thiazide), as we wished not to exclude those who had progressed from a thiazide to a loop; c) prescription for a potassium supplement in the baseline period; d) cancer diagnosis in the baseline period; e) occurrence of an outcome of interest in the baseline period; or f) age <18 or ≥95 years. The beginning of the course was defined by the fill date of the beneficiary's first loop diuretic prescription. The end of the course was defined by the first occurrence of the following: a) an outcome of interest; b) death, for the study of SD/VA; c) a cancer diagnosis; d) a switch to another class of diuretics; e) a >60-day gap between consecutive loop diuretic prescriptions; or f) the end of follow-up time in the database. Incident courses ≤2 days in length were excluded, as this was the time period over which exposure to empiric potassium supplementation was assessed (see below) and therefore such courses provided no follow-up time. For the 96% of subjects whose initial loop diuretic was furosemide, we stratified the death analyses by initial furosemide dose, expressed as <40 mg/day vs. ≥40 mg/day.

### Ascertainment of exposure and covariates

The exposure of interest was empiric potassium supplementation, defined as filling a prescription for potassium (as a solid bicarbonate, chloride, citrate, or gluconate salt—and excluding from study persons having received a liquid preparation based on the aforementioned rationale) on the same day as or on the day following the initial loop diuretic prescription. We did not examine potassium dispensed ≥2 days following initiation of loop diuretic therapy because such potassium prescriptions were likely to represent responses to clinical or laboratory signs of hypokalemia (i.e., reactive supplementation) rather than clinical decisions to supplement empirically. Those without empiric potassium supplementation served as the reference group.

We measured three types of potential confounders: 1) demographics—age, sex, race, state of residence, calendar year, dual-eligibility status, and nursing home residence status; 2) diseases, measured as ever prior to the initial loop course—such as chronic illnesses (e.g., diabetes mellitus, chronic kidney disease), potential risk factors for death or SD/VA (e.g., heart failure), labeled and off-labeled indications for diuretics or potassium supplementation (e.g., ascites, metabolic alkalosis), and contraindications for receiving potassium supplementation (e.g., acidosis); and 3) drug markers of chronic disease, measured as ever prior to the initial loop course (e.g., insulin as a marker for diabetes mellitus). Table 1 lists all measured covariates.

**Table 1:** Baseline characteristics of beneficiaries in the primary outcome (all-cause death) cohort, before and after propensity score matching.

	OVERALL LOOP DIURETIC COHORT						FUROSEMIDE <40 MG/DAY COHORT						FUROSEMIDE ≥40 MG/DAY COHORT					
	Before PS Matching			After 1-to-1 PS Matching			Before PS Matching			After 1-to-1 PS Matching			Before PS Matching			After 1-to-1 PS Matching		
Covariate	K^+^ = YES	K^+^ = NO	SDiff	K^+^ = YES	K^+^ = NO	SDiff	K^+^ = YES	K^+^ = NO	SDiff	K^+^ = YES	K^+^ = NO	SDiff	K^+^ = YES	K^+^ = NO	SDiff	K^+^ = YES	K^+^ = NO	SDiff
	n = 179,436	n = 474,624		n = 179,425	n = 179,425		n = 71,631	n = 243,436		n = 71,631	n = 71,631		n = 101,704	n = 210,460		n = 100,869	n = 100,869	
**DEMOGRAPHICS**																		
Age, in years		0.05		<0.01		0.09		<0.01		0.03		<0.01						
18–44	14.3%	15.5%	—	14.3%	14.1%	—	14.4%	16.4%	—	14.4%	14.6%	—	14.3%	14.6%	—	14.3%	13.9%	—
45–54	14.1%	14.4%	—	14.1%	14.2%	—	13.0%	13.8%	—	13.0%	12.8%	—	14.9%	15.1%	—	14.9%	14.9%	—
55–64	15.0%	15.6%	—	15.0%	14.9%	—	13.5%	14.4%	—	13.5%	13.4%	—	16.2%	17.0%	—	16.2%	16.2%	—
65–74	20.6%	20.7%	—	20.6%	20.6%	—	19.9%	20.1%	—	19.9%	19.9%	—	21.2%	21.2%	—	21.2%	21.4%	—
75–84	22.3%	21.3%	—	22.3%	22.4%	—	23.4%	21.6%	—	23.4%	23.5%	—	21.4%	20.7%	—	21.4%	21.6%	—
85–94	13.7%	12.6%	—	13.7%	13.8%	—	15.8%	13.7%	—	15.8%	15.7%	—	12.0%	11.3%	—	12.0%	12.0%	—
Sex, female	71.0%	70.0%	0.03	71.0%	71.2%	<0.01	73.8%	72.9%	0.02	73.8%	73.7%	<0.01	68.8%	66.2%	0.06	68.8%	68.9%	<0.01
Race																		
white	54.6%	49.6%	0.10	54.6%	54.8%	<0.01	54.3%	49.5%	0.10	54.3%	54.3%	<0.01	54.2%	48.6%	0.11	54.0%	54.2%	<0.01
black	15.6%	17.5%	0.05	15.6%	15.4%	<0.01	13.2%	15.6%	0.06	13.2%	13.3%	<0.01	17.3%	19.9%	0.07	17.4%	17.2%	<0.01
hispanic	15.4%	18.1%	0.07	15.4%	15.4%	<0.01	16.1%	18.6%	0.07	16.1%	16.1%	<0.01	15.1%	17.8%	0.07	15.1%	15.0%	<0.01
other/unknown	14.4%	14.8%	0.01	14.4%	14.5%	<0.01	16.3%	16.4%	<0.01	16.3%	16.3%	<0.01	13.5%	13.7%	<0.01	13.5%	13.6%	<0.01
Medicaid-Medicare dual eligibility status, yes	69.9%	67.4%	0.05	69.9%	70.0%	<0.01	70.7%	66.2%	0.10	70.7%	70.7%	<0.01	68.9%	68.0%	0.02	68.9%	69.0%	<0.01
Nursing home residence status, yes	15.4%	14.6%	0.02	15.4%	15.4%	<0.01	16.8%	15.1%	0.05	16.8%	17.0%	<0.01	13.9%	13.7%	<0.01	13.9%	14.0%	<0.01
**COHORT-DEFINING CHARACTERISTICS**																		
Cohort-initiating loop: bumetanide*	1.8%	2.1%	—	1.8%	2.3%	—	0.0%	0.0%	—	0.0%	0.0%	—	0.0%	0.0%	—	0.0%	0.0%	—
Cohort-initiating loop: ethacrynic acid*	0.0%	0.1%	—	0.0%	0.1%	—	0.0%	0.0%	—	0.0%	0.0%	—	0.0%	0.0%	—	0.0%	0.0%	—
Cohort-initiating loop: furosemide*	96.6%	95.7%	—	96.6%	95.3%	—	100%	100%	—	100%	100%	—	100%	100%	—	100%	100%	—
Starting dose <40 mg/day*	41.5%	53.8%	—	41.3%	54.5%	—	100%	100%	—	100%	100%	—	—	—	—	—	—	—
Starting dose = 40 mg/day*	47.0%	35.4%	—	47.2%	35.1%	—	—	—	—	—	—	—	80.5%	76.7%	—	80.4%	80.4%	—
Starting dose >40 mg/day*	11.5%	10.8%	—	11.4%	10.3%	—	—	—	—	—	—	—	19.5%	23.3%	—	19.6%	19.6%	—
Cohort-initiating loop: torsemide*	1.6%	2.2%	—	1.6%	2.3%	—	0.0%	0.0%	—	0.0%	0.0%	—	0.0%	0.0%	—	0.0%	0.0%	—
**EXPOSURE-DEFINING AND RELATED CHARACTERISTICS**																		
K^+^ at any time during follow-up*	100%	13.5%	—	100%	15.7%	—	100%	11.6%	—	100%	13.6%	—	100%	15.7%	—	100%	18.2%	—
K^+^ during the entirety of follow-up*	46.3%	0%	—	46.3%	0%	—	49.0%	0.0%	—	49.0%	0%	—	44.7%	0%	—	44.7%	0%	—
Proportion of days on K^+^ (mean, standard deviation)*	0.849 (0.23)	0.067 (0.20)	—	0.849 (0.23)	0.079 (0.21)	—	0.858 (0.23)	0.056 (0.18)	—	0.858 (0.23)	0.067 (0.20)	—	0.842 (0.23)	0.079 (0.21)	—	0.841 (0.23)	0.093 (0.23)	—
Proportion of days on K^+^ (median, IQR)*	0.98 (0.77–1.00)	0.00 (0.00–0.00)	—	0.98 (0.77–1.00)	0.00 (0.00–0.00)	—	0.99 (0.80– 1.00)	0.00 (0.00– 0.00)	—	0.99 (0.80–1.00)	0.00 (0.00–0.00)	—	0.97 (0.75–1.00)	0.00 (0.00–0.00)	—	0.97 (0.75–1.00)	0.00 (0.00–0.00)	—
Lab monitoring of K^+^ in first 30 days of loop prescription*	35.9%	32.2%	—	34.6%	31.3%	—	35.0%	30.6%	—	36.6%	33.7%	—	35.5%	32.1%	—	35.5%	31.4%	—
**DISEASES, EVER PRIOR TO INITIAL LOOP COURSE**																		
Acidosis	2.1%	2.5%	0.03	2.1%	2.0%	<0.01	1.9%	2.0%	<0.01	1.9%	1.8%	<0·01	2.2%	3.2%	0.06	2.2%	2.1%	<0.01
Alcohol abuse**	6.3%	6.6%	0.01	6.3%	6.2%	<0.01	5.9%	6.6%	0.03	5.9%	5.8%	<0.01	6.6%	6.8%	0.01	6.5%	6.4%	<0.01
Alkalosis, metabolic	0.5%	0.5%	<0.01	0.5%	0.5%	<0.01	0.5%	0.4%	<0.01	0.5%	0.5%	<0.01	0.5%	0.6%	0.01	0.5%	0.5%	<0.01
Amyloidosis	0.1%	0.1%	<0.01	0.1%	0.1%	<0.01	0.1%	0.1%	<0.01	0.1%	0.1%	<0.01	0.1%	0.1%	0.02	0.1%	0.1%	<0.01
Anemia	43.2%	43.1%	<0.01	43.2%	43.0%	<0.01	44.5%	42.2%	0.05	44.5%	44.3%	<0.01	42.2%	43.9%	0.03	42.1%	41.8%	<0.01
Arrhythmia/conduction disorder	36.0%	33.1%	0.06	36.0%	36.0%	<0.01	35.0%	31.6%	0.07	35.0%	34.9%	<0.01	36.4%	34.6%	0.04	36.3%	36.2%	<0.01
Ascites	1.6%	2.1%	0.03	1.6%	1.6%	<0.01	1.5%	1.6%	0.01	1.5%	1.5%	<0.01	1.8%	2.6%	0.05	1.8%	1.8%	<0.01
Asthma/COPD	44.9%	39.7%	0.11	44.9%	44.8%	<0.01	44.0%	39.4%	0.09	44.0%	43.9%	<0·01	45.4%	39.9%	0.11	45.2%	44.9%	<0.01
Cerebrovascular disease	30.8%	28.8%	0.04	30.8%	30.8%	<0.01	31.7%	28.7%	0.06	31.7%	31.5%	<0.01	29.9%	28.6%	0.03	29.7%	29.7%	<0.01
Coronary artery disease	48.8%	45.2%	0.07	48.8%	48.8%	<0.01	47.0%	42.9%	0.08	47.0%	46.8%	<0.01	49.8%	47.4%	0.05	49.5%	49.2%	<0.01
Corticoadrenal insufficiency	0.9%	1.0%	0.01	0.9%	0.9%	<0.01	1.0%	1.1%	<0.01	1.0%	1.0%	<0.01	0.9%	1.0%	0.01	0.9%	0.9%	<0.01
Cushing's syndrome	0.2%	0.2%	<0.01	0.2%	0.2%	<0.01	0.2%	0.2%	<0.01	0.2%	0.2%	<0.01	0.2%	0.2%	<0.01	0.2%	0.2%	<0.01
Diabetes insipidus	0.1%	0.2%	<0.01	0.1%	0.1%	<0.01	0.1%	0.2%	0.01	0.1%	0.1%	<0.01	0.1%	0.2%	<0.01	0.1%	0.1%	<0.01
Diabetes mellitus	45.3%	47.6%	0.05	45.3%	45.2%	<0.01	42.7%	44.7%	0.04	42.7%	42.4%	<0.01	46.9%	50.7%	0.08	47.0%	46.9%	<0.01
Edema	27.0%	25.2%	0.04	27.0%	27.1%	<0.01	27.6%	24.5%	0.07	27.6%	27.5%	<0.01	26.2%	25.4%	0.02	26.1%	26.0%	<0.01
Glaucoma	14.3%	14.9%	0.02	14.3%	14.3%	<0.01	14.9%	15.4%	0.01	14.9%	14.7%	<0.01	13.8%	14.2%	0.01	13.8%	13.8%	<0.01
Heart failure/cardiomyopathy	42.2%	39.2%	0.06	42.2%	42.2%	<0.01	37.6%	33.8%	0.08	37.6%	37.3%	<0.01	45.2%	45.1%	<0.01	45.1%	44.7%	<0.01
HIV/AIDS	1.8%	3.1%	0.08	1.8%	1.7%	<0.01	1.8%	3.3%	0.09	1.8%	1.7%	<0.01	1.7%	3.1%	0.09	1.8%	1.7%	<0.01
Hypercholesterolemia	52.9%	50.5%	0.05	52.9%	52.8%	<0.01	53.1%	50.9%	0.04	53.1%	52.7%	<0.01	52.7%	49.8%	0.06	52.5%	52.3%	<0.01
Hyperkalemia	3.9%	7.1%	0.13	3.9%	3.8%	<0.01	3.6%	5.5%	0.09	3.6%	3.5%	<0.01	4.1%	8.7%	0.18	4.1%	3.7%	0.02
Hyperosmolality	1.4%	1.6%	0.02	1.4%	1.3%	<0.01	1.5%	1.5%	<0.01	1.5%	1.5%	<0.01	1.2%	1.6%	0.03	1.3%	1.3%	<0.01
Hypertension	75.3%	74.9%	<0.01	75.3%	75.2%	<0.01	74.0%	73.1%	0.02	74.0%	73.7%	<0.01	76.0%	76.6%	0.01	75.9%	75.6%	<0.01
Hyperthyroidism	5.5%	5.2%	0.01	5.5%	5.5%	<0.01	5.9%	5.5%	0.02	5.9%	5.8%	<0.01	5.2%	4.9%	0.02	5.2%	5.2%	<0.01
Hypokalemia	10.4%	8.3%	0.07	10.4%	10.2%	<0.01	10.3%	7.7%	0.09	10.3%	10.0%	<0.01	10.3%	8.8%	0.05	10.1%	10.0%	<0.01
Hypothyroidism	25.1%	22.6%	0.06	25.1%	25.2%	<0.01	26.3%	23.3%	0.07	26.3%	26.1%	<0.01	24.3%	21.5%	0.07	24.2%	24.1%	<0.01
Kidney disease	24.3%	30.0%	0.13	24.3%	24.1%	<0.01	23.7%	26.3%	0.06	23.7%	23.5%	<0.01	24.5%	33.8%	0.20	24.6%	24.4%	<0.01
Liver disease	17.0%	17.6%	0.02	17.0%	16.9%	<0.01	17.4%	17.3%	<0.01	17.4%	17.2%	<0.01	16.8%	18.0%	0.03	16.8%	16.6%	<0.01
Mg^2+^ metabolism disorder	1.8%	1.8%	<0.01	1.8%	1.7%	<0.01	1.7%	1.6%	0.01	1.7%	1.7%	<0.01	1.7%	2.1%	0.02	1.7%	1.7%	<0.01
Nocturia	2.1%	2.0%	0.01	2.1%	2.2%	<0.01	2.3%	2.2%	0.01	2.3%	2.3%	<0.01	2.0%	1.8%	0.02	2.0%	1.9%	<0.01
Obesity**	18.0%	16.1%	0.05	18.0%	17.9%	<0.01	15.6%	15.3%	<0.01	15.6%	15.6%	<0.01	19.6%	16.9%	0.07	19.4%	19.3%	<0.01
Pulmonary circulation	7.8%	7.0%	0.03	7.8%	7.7%	<0.01	7.0%	6.0%	0.04	7.0%	6.9%	<0.01	8.2%	8.0%	<0.01	8.1%	8.1%	<0.01
Pulmonary congestion and hypostasis/pulmonary edema	11.5%	10.3%	0.04	11.5%	11.5%	<0.01	10.6%	8.8%	0.06	10.6%	10.5%	<0.01	12.0%	12.0%	<0.01	11.9%	11.8%	<0.01
Pyloric stenosis	0.2%	0.2%	<0.01	0.2%	0.2%	<0.01	0.2%	0.2%	<0.01	0.2%	0.2%	<0.01	0.2%	0.2%	<0.01	0.2%	0.2%	<0.01
Rheumatoid arthritis and other inflammatory polyarthropathies	27.8%	25.6%	0.05	27.8%	27.8%	<0.01	28.6%	26.8%	0.04	28.6%	28.3%	<0.01	27.5%	24.2%	0.07	27.3%	27.3%	<0.01
Sickle cell disease	0.2%	0.2%	0.01	0.2%	0.2%	<0.01	0.2%	0.3%	0.02	0.2%	0.2%	<0.01	0.2%	0.2%	0.02	0.2%	0.2%	<0.01
Smoking, tobacco**	15.0%	12.4%	0.08	15.0%	14.9%	<0.01	14.6%	12.3%	0.07	14.6%	14.8%	<0.01	15.4%	12.6%	0.08	15.2%	14.8%	<0.01
Substance abuse**	5.6%	6.2%	0.03	5.6%	5.5%	<0.01	5.4%	6.2%	0.03	5.4%	5.4%	<0.01	5.8%	6.3%	0.02	5.8%	5.6%	<0.01
Systemic lupus erythematosus	1.3%	1.3%	<0.01	1.3%	1.3%	<0.01	1.2%	1.3%	<0.01	1.2%	1.2%	<0.01	1.3%	1.4%	<0.01	1.3%	1.2%	<0.01
Uropathy, obstructive	0.1%	0.1%	<0.01	0.1%	0.1%	<0.01	0.1%	0.1%	<0.01	0.1%	0.1%	<0.01	0.1%	0.1%	<0.01	0.1%	0.1%	<0.01
Valvular heart disease	28.5%	24.4%	0.09	28.4%	28.4%	<0.01	27.4%	22.9%	0.10	27.4%	27.4%	<0.01	28.9%	25.8%	0.07	28.6%	28.4%	<0.01
**DRUGS MARKER OF CHRONIC DISEASE, EVER PRIOR TO INITIAL LOOP COURSE**																		
ACEIs/ATIIRBs	43.4%	47.1%	0.07	43.4%	43.4%	<0.01	43.0%	47.0%	0.08	43.0%	42.9%	<0.01	44.2%	47.7%	0.07	44.2%	44.0%	<0.01
Adrenergic bronchodilators	33.6%	30.5%	0.07	33.6%	33.4%	<0.01	34.8%	32.6%	0.05	34.8%	34.8%	<0.01	33.1%	28.4%	0.10	32.8%	32.5%	<0.01
Anorexiants/antiobesity agents	0.4%	0.3%	0.02	0.4%	0.4%	<0.01	0.3%	0.3%	0.01	0.3%	0.3%	<0.01	0.4%	0.2%	0.03	0.4%	0.4%	<0.01
Antiadrenergic agents	6.6%	7.5%	0.04	6.6%	6.5%	<0.01	6.4%	6.8%	0.02	6.4%	6.2%	<0.01	6.8%	8.5%	0.06	6.9%	6.7%	<0.01
Antiarrhythmics, type I, except lidocaine and phenytoin	0.7%	0.6%	0.02	0.7%	0.7%	<0.01	0.8%	0.6%	0.02	0.8%	0.8%	<0.01	0.7%	0.6%	0.01	0.7%	0.7%	<0.01
Antiarrhythmics, type III	2.0%	1.8%	0.02	2.0%	2.1%	<0.01	2.0%	1.7%	0.02	2.0%	2.0%	<0.01	2.1%	1.9%	0.01	2.1%	2.1%	<0.01
Antidiabetics	27.5%	30.9%	0.07	27.5%	27.6%	<0.01	25.7%	29.3%	0.08	25.7%	25.6%	<0.01	28.9%	33.0%	0.09	29.0%	29.0%	<0.01
Antiglaucoma agents, ophthalmic	7.2%	7.6%	0.01	7.2%	7.2%	<0.01	7.7%	8.0%	<0.01	7.7%	7.6%	<0.01	6.8%	7.1%	0.01	6.9%	6.7%	<0.01
Antiglaucoma agents, oral	0.5%	0.6%	<0.01	0.5%	0.5%	<0.01	0.5%	0.6%	<0.01	0.5%	0.5%	<0.01	0.5%	0.5%	<0.01	0.5%	0.5%	<0.01
Antihyperlipidemics	34.6%	35.1%	0.01	34.6%	34.5%	<0.01	35.4%	36.4%	0.02	35.4%	35.2%	<0.01	34.3%	33.9%	<0.01	34.2%	34.1%	<0.01
Antiretrovirals	0.8%	1.3%	0.05	0.8%	0.7%	<0.01	0.8%	1.3%	0.05	0.8%	0.7%	<0.01	0.8%	1.3%	0.05	0.8%	0.7%	<0.01
Beta blockers, systemic	30.4%	31.5%	0.02	30.4%	30.3%	<0.01	31.5%	32.3%	0.02	31.5%	31.5%	<0.01	29.9%	30.9%	0.02	29.9%	29.7%	<0.01
Bisphosphonates	11.0%	9.9%	0.04	11.0%	10.9%	<0.01	13.4%	11.7%	0.05	13.4%	13.2%	<0.01	9.3%	7.8%	0.05	9.3%	9.2%	<0.01
Calcium channel blockers, non-verapamil	31.9%	33.7%	0.04	31.9%	31.6%	<0.01	32.2%	33.5%	0.03	32.2%	31.9%	<0.01	32.0%	34.3%	0.05	32.0%	31.5%	0.01
Calcium channel blocker, verapamil	4.9%	4.6%	0.02	4.9%	4.9%	<0.01	4.8%	4.6%	0.01	4.8%	4.7%	<0.01	5.0%	4.5%	0.02	4.9%	4.9%	<0.01
Corticosteroids, inhaled	15.8%	14.1%	0.05	15.8%	15.7%	<0.01	16.4%	15.2%	0.03	16.4%	16.3%	<0.01	15.5%	13.0%	0.07	15.4%	15.2%	<0.01
Corticosteroids, oral	22.2%	20.1%	0.05	22.2%	22.0%	<0.01	23.4%	21.7%	0.04	23.4%	23.3%	<0.01	21.5%	18.3%	0.08	21.3%	20.9%	<0.01
Digoxin	7.1%	6.4%	0.03	7.1%	7.1%	<0.01	7.3%	6.2%	0.05	7.3%	7.3%	<0.01	7.0%	6.8%	0.01	7.0%	7.0%	<0.01
Diuretics, thiazides*	32.6%	31.0%	—	32.6%	30.1%	—	32.3%	31.7%	—	32.3%	30.5%	—	33.1%	30.2%	—	33.1%	30.2%	—
Immunosuppressives	0.4%	1.1%	0.07	0.4%	0.4%	0.01	0.4%	0.8%	0.05	0.4%	0.4%	<0.01	0.4%	1.4%	0.09	0.4%	0.4%	<0.01
Nitrates	19.9%	18.2%	0.05	19.9%	19.9%	<0.01	20.1%	18.4%	0.04	20.1%	20.0%	<0.01	20.0%	18.0%	0.05	19.8%	19.8%	<0.01
Thyroid hormones	11.3%	10.8%	0.02	11.3%	11.3%	<0.01	12.3%	11.5%	0.03	12.3%	12.3%	<0.01	10.7%	9.9%	0.03	10.6%	10.6%	<0.01
Vasodilators, non-nitrates	0.9%	1.6%	0.06	0.9%	0.9%	<0.01	0.8%	1.1%	0.03	0.8%	0.8%	<0.01	1.0%	2.2%	0.09	1.0%	0.9%	0.01
Warfarin	8.2%	7.8%	0.01	8.2%	8.2%	<0.01	8.1%	7.6%	0.02	8.1%	8.1%	<0.01	8.2%	8.0%	<0.01	8.2%	8.2%	<0.01
Xanthine derivatives	4.9%	3.9%	0.05	4.9%	4.8%	<0.01	4.9%	4.1%	0.04	4.9%	4.9%	<0.01	4.9%	3.7%	0.06	4.8%	4.7%	<0.01

* not included in the propensity score.

** health-related behavior or state ascertained via diagnostic codes alone.

PS: propensity score; K^+^: empiric potassium supplementation; SDiff: standardized difference; IQR = interquartile range; COPD: chronic obstructive pulmonary disease; HIV: human immunodeficiency virus; AIDS: acquired immunodeficiency syndrome; Mg^2+^: magnesium; ACEIs: angiotensin-converting enzyme inhibitors; ATIIRBs: angiotensin-II receptor blocker.

For the overall group of loop diuretic users, and for furosemide users stratified by initial furosemide dose, we performed propensity score matching using one-to-one, nearest neighbor matching (caliper width = 10% of the standard deviation of the logit of the propensity score [13]) without replacement. Propensity scores were calculated by logistic regression using the variables listed in Table 1. Age was modeled using splines [14].

### Study Outcomes

The primary outcome was all-cause death. The secondary outcome was outpatient-originating SD/VA resulting in emergency department or hospital presentation. The rationale for considering SD/VA as a composite outcome is that sudden cardiac death is often due to undocumented ventricular arrhythmia [15]. Incident SD/VA outcomes were identified in emergency department and inpatient claims having one of the following International Classification of Diseases (ICD-9) discharge diagnoses in a first-listed or principal position: paroxysmal ventricular tachycardia (427.1), ventricular fibrillation and flutter (427.4), ventricular fibrillation (427.41), ventricular flutter (427.42), cardiac arrest (427.5), sudden death (798), instantaneous death (798.1), or death occurring in <24 hours from onset of symptoms, not otherwise explained (798.2). This algorithm has a positive predictive value of 85% for identifying outpatient-originating SD/VA not due to extrinsic causes [16], [17].

### Statistical analyses

We first compared baseline characteristics of the cohorts before and after propensity score matching. We evaluated baseline differences by calculating standardized mean differences, using a threshold of 0.10 to indicate potential imbalance [18]. We next calculated incidence rates (with 95% confidence intervals [CIs]) for each outcome, stratified by empiric potassium use. We then plotted time-to-event curves by furosemide dose. Finally, we fitted stratified Cox proportional hazards models, which account for matching, to obtain estimated hazard ratios (HRs) for the associations between empiric potassium supplementation and the outcome within furosemide dose strata. Sub-analyses stratified by furosemide dose examined potential effect modification by: age; history of arrhythmia/conduction disorder in the 12 months prior to the loop course; history of kidney disease (an algorithm for which has an expected sensitivity of ∼80% [19]) in the 12 months prior to the loop course; potassium laboratory monitoring within 30 days following the loop course; and initial empiric potassium dose. The potassium laboratory monitoring subgroup analysis excluded deaths occurring in the first 30 days in order to minimize immortal time bias. Analyses were conducted using SAS v9.3 (SAS Institute Inc.: Cary, NC).

## Results

### Cohort composition

We identified 654,060 persons who met eligibility criteria and initiated therapy with a loop diuretic (Table 1). Greater than 70% were female and about 50% were white; the mean age of these individuals was about 65 years. Within this cohort, 27% received empiric potassium supplementation (N = 179,436) and 73% did not (N = 474,624). The proportions of persons receiving empiric potassium supplementation differed between users of furosemide <40 mg/day and ≥40 mg/day, at 23% and 33% respectively. The proportion of follow-up days covered by an active potassium prescription was 0.849 in the empiric supplementation group and 0.079 in the reference group.

When comparing users of loop diuretics at any dose by exposure status, only three measured baseline factors were potentially unevenly distributed—prior history of: asthma/chronic obstructive pulmonary disease; hyperkalemia; and kidney disease. We found an acceptable match for all but 11 potassium-exposed persons (N = 179,425) and therefore the underlying study cohort consisted of 358,850 loop users—164,833 in the furosemide <40 mg/day cohort and 179,439 in the furosemide ≥40 mg/day cohort. After propensity score matching, all of the standardized mean differences were <0.02 (Table 1), suggesting that propensity score matching resulted in very similar distributions of measured covariates by exposure group.

### Primary outcome: all-cause death

**Figure 1:**
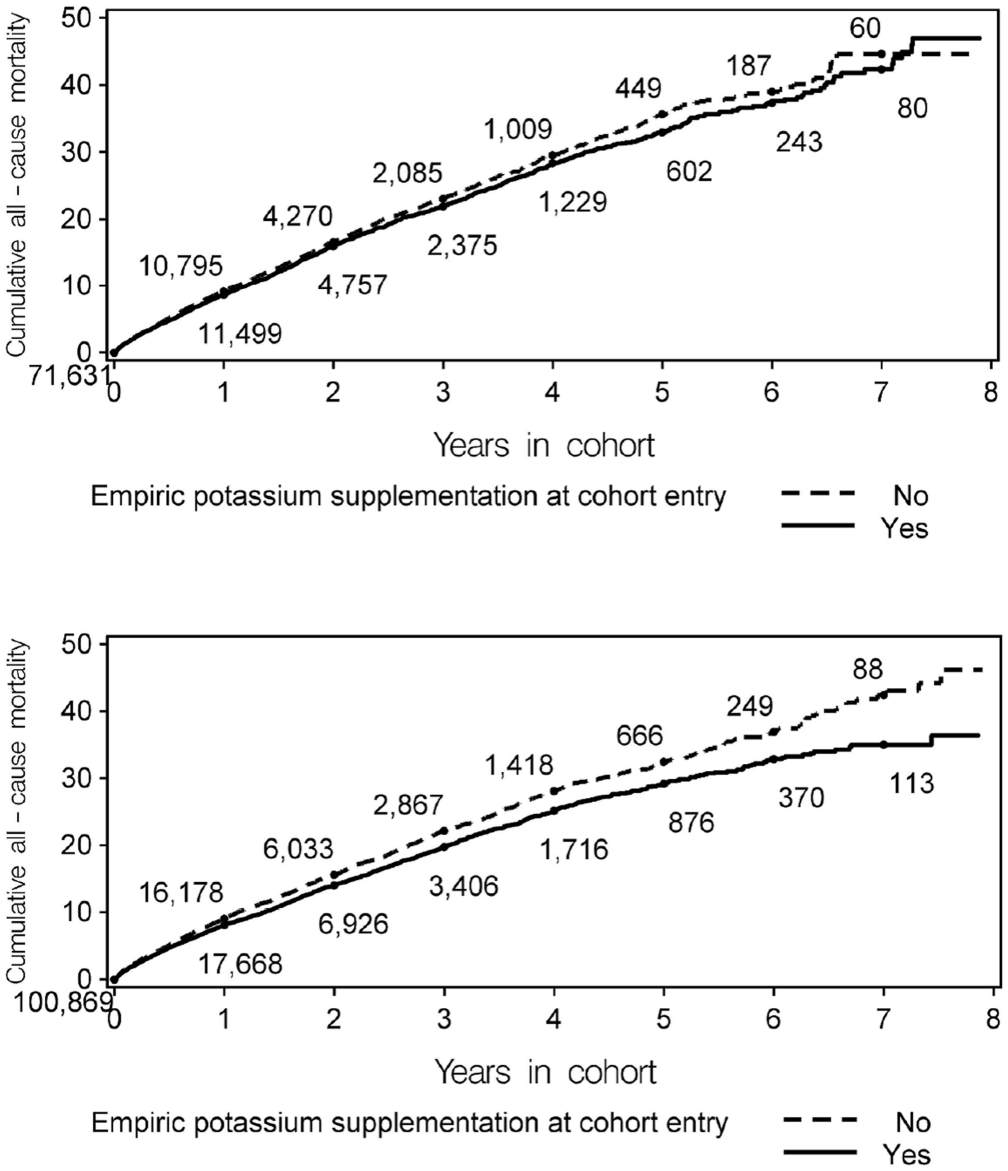
Time-to-death curves for empiric potassium supplementation among furosemide initiators, stratified by initial furosemide dose. Figure 1a. Initial furosemide dose <40 mg/day (N = 71,631 in each of the empiric potassium exposed and unexposed groups). Figure 1b. Initial furosemide dose ≥40 mg/day (N = 100,869 in each of the empiric potassium exposed and unexposed groups).

In the overall loop user cohort, we identified 31,653 deaths for a mortality rate of 90.7 per 1,000 p-y (95% CI: 89.7 to 91.7). Mortality rates were 88.5 (95% CI: 87.0 to 89.9) and 91.6 (95% CI: 90.2 to 93.1) per 1,000 p-y in furosemide <40 mg/day and ≥40 mg/day cohorts respectively. The overall crude and matched HRs for empiric potassium supplementation and all-cause death were 1.02 (95% CI: 0.99 to 1.06, p = 0.189) and 0.93 (95% CI: 0.89 to 0.98, p = 0.003), respectively. Among those whose initial furosemide dose was <40 mg/day, the crude and matched HRs for empiric potassium supplementation were 1.12 (95% CI: 1.07 to 1.18, p<0.0001) and 0.93 (95% CI: 0.86 to 1.00, p = 0.050), respectively. Among those whose initial furosemide dose was ≥40 mg/day, the crude and matched HRs for empiric potassium were 0.93 (95% CI: 0.89 to 0.97, p = 0.002) and 0.84 (95% CI: 0.79 to 0.89, p<0.0001), respectively. The time-to-event curves, in the matched cohorts, for receiving potassium supplementation vs. not in those whose initial furosemide dose was ≥40 mg/day began to diverge during the first year; see Figure 1.

To further examine whether the apparent benefit of potassium increased monotonically with furosemide dose, we separately calculated matched HRs for empiric potassium in those whose initial furosemide dose was exactly 40 mg/day and >40 mg/day, yielding matched HRs of 0.84 (95% CI: 0.78 to 0.90, p<0.0001) and 0.83 (95% CI: 0.74 to 0.93, p = 0.001) respectively. Thus, monotonicity was observed across three levels (<40,  = 40, and >40 mg) of daily furosemide dose.

**Figure 2:**
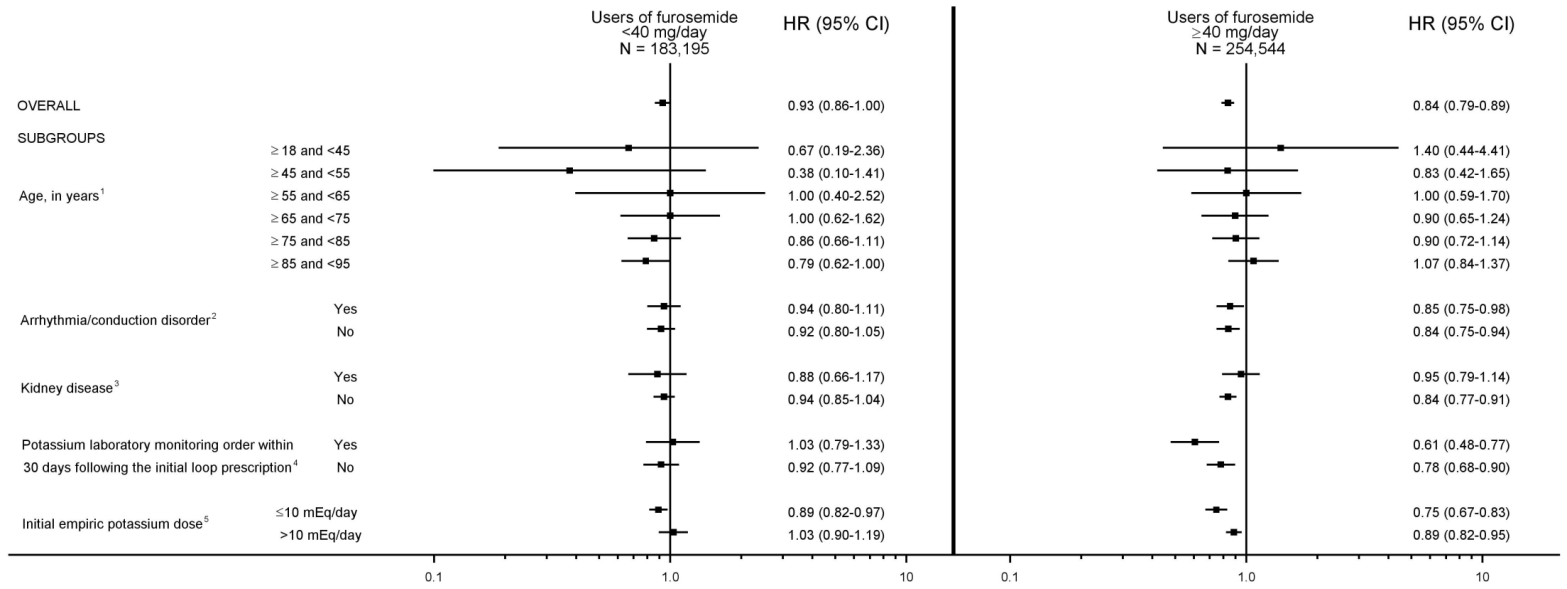
Risk of death for empiric potassium supplementation vs. no empiric potassium supplementation among furosemide initiators: propensity score-matched analyses examining patient subgroups. HR = hazard ratio; CI = confidence interval. p-values for the difference in effect estimates within stratum, in users of furosemide <40 mg/day and in users of furosemide ≥40 mg/day. 1, p = 0.86 and p = 0.24, respectively. 2, p = 0.75 and p = 0.81, respectively. 3, p = 0.66 and p = 0.49, respectively. 4, p = 0.74 and p = 0.37, respectively. 5, p = 0.08 and p<0.01, respectively.

Figure 2 shows the results of subgroup analyses stratified on initial furosemide dose. Of note, for the subgroup analyses of age, arrhythmia/conduction disorder, kidney disease, and potassium laboratory monitoring, all of the standardized mean differences for covariates by exposure status were <0.10 after propensity score matching (data not shown). For the subgroup analysis examining potassium dose, while the vast majority of post-propensity score matching standardized mean differences were <0.10, the maximum was 0.20 (see Appendix S1).

A statistically significant difference in the association between empiric potassium supplementation and death was observed in the underlying cohort of furosemide ≥40 mg/day users within the following subgroup: initial empiric potassium dose ≤10 mEq/day vs. >10 mEq/day (HRs = 0.75 vs. 0.89, p-value for difference <0.01).

### Secondary outcome: sudden cardiac death/ventricular arrhythmia

Among 629,949 incident courses of loop diuretic use, we identified 1,470 incident occurrences of SD/VA for an incidence rate of 4.5 per 1,000 p-y (95% CI: 4.2 to 4.7) within the overall loop user cohort. The overall crude and matched HRs for empiric potassium supplementation and SD/VA were 1.13 (95% CI: 1.01 to 1.26, p = 0.030) and 1.02 (95% CI: 0.83 to 1.24, p = 0.879), respectively.

## Discussion

In new initiators of loop diuretics, empiric potassium supplementation was associated with a reduction in all-cause mortality. The relative reduction was 7% and at the threshold for statistical significance (p = 0.05) in those whose initial furosemide dose was <40 mg/day, and 16% and statistically significant in those whose initial furosemide dose was ≥40 mg/day. Monotonicity was observed across three levels of furosemide dose, and the apparent benefit of potassium in persons receiving ≥40 mg/day of furosemide was observable within the first year. Taken together, these results suggest that empiric potassium supplementation may be associated with improved survival in those receiving a loop diuretic, and that the degree of benefit increases with diuretic dose. Given that mortality was about 9% per year in this cohort, the magnitude of the absolute benefit is substantial, especially in those receiving higher doses of furosemide. The estimated number of patients needed to be empirically-supplemented with potassium (i.e., number needed to treat) [20] to prevent one death within the first year after initiating furosemide <40 mg/day,  = 40 mg/day, and >40 mg/day is 164, 72, and 67, respectively.

In a subgroup analysis of persons with no history of kidney disease receiving ≥40 mg/day of furosemide, empiric potassium supplementation was associated with a 16% reduction in death, an association not evident among persons with existing kidney disease. The latter is not surprising, given that persons with renal impairment may be at increased risk for hyperkalemia, which may negate potassium's otherwise beneficial effect [21], [22]. Further, a 25% reduction in mortality among users of furosemide ≥40 mg/day was limited to persons receiving empiric potassium doses of ≤10 mEq/day. Whether this is indicative of the optimal dosage range of empiric potassium supplementation or due to confounding (i.e., because propensity score matching was not designed to balance covariates between potassium dose strata) deserves further elucidation.

To our knowledge, this is the first study designed to examine the association between empiric potassium supplementation and rates of clinical outcomes in new initiators of loop diuretics. Earlier studies have found no effect of potassium supplementation on the risk of either laboratory-defined hypokalemia or clinical outcomes [23]–[25], but these studies examined either thiazide users alone or included together with users of loop diuretics. An additional study examined clinical outcomes associated with baseline potassium use in patients with heart failure [26], but included patients receiving and not receiving diuretics, did not begin follow-up with the initiation of a diuretic, and did not stratify on diuretic dose.

Given that one major mechanism by which potassium may improve survival is reduction in the risk of serious ventricular arrhythmia caused by potassium depletion, it was surprising that potassium did not appear to reduce the risk of SD/VA. However, our finding is consistent with a retrospective analysis of trial data [27] in which potassium supplementation did not affect the incidence of arrhythmic death (p = 0.4) among persons with left ventricular dysfunction [28].

Strengths of this study include its large sample size, unambiguous primary outcome measure, similarity of compared groups even before matching, restriction to new starters of loop diuretics, examination of empiric rather than reactive potassium supplementation, and stratification by furosemide dose.

This study has limitations. First, because of the design, the effect of reactive potassium supplementation was not examined. Second, despite our demonstration of covariate balance between the exposure groups both pre- and post-propensity score adjustment, there exists the potential for residual confounding by unmeasured or poorly-measured variables and/or behaviors. In particular, it is possible that persons with mild renal insufficiency may be channeled away from potassium supplementation and may be at higher risk for death than baseline. Arguing against this possibility are findings that mild-to-moderate renal insufficiency may not be an independent risk factor for death [29], [30]. Regardless, we controlled for the presence of diagnosed chronic kidney disease, codes for which may have a sensitivity as high as 80% [19]. Third, we were unable to capture magnesium supplement exposures due to their typical use over-the-counter. An additional limitation includes the potential insensitivity of the SD/VA diagnoses and wide confidence intervals in subgroup analyses.

In conclusion, this study provides evidence that the strategy of initiating potassium supplementation together with loop diuretic therapy appears to be associated with increased survival, and that the degree of benefit increases with increasing diuretic dose. Because of the importance of this question, these results deserve to be replicated.

## Supporting Information

Appendix S1 **Baseline characteristics of beneficiaries in the subgroup analysis examining potassium supplementation dose and all-cause death, before and after propensity score matching on potassium exposure status.**
(DOCX)
