# Supplementary material for: Empiric Potassium Supplementation and Increased Survival in Users of Loop Diuretics
Source: PLoS One. 2014 Jul 16;9(7):e102279. doi: 10.1371/journal.pone.0102279 (PMC4100893; doi:10.1371/journal.pone.0102279)
Supplement: Appendix S1 — Baseline characteristics of beneficiaries in the subgroup analysis examining potassium supplementation dose and all-cause death, before and after propensity score matching on potassium exposure status. (DOCX) [file pone.0102279.s001.docx]

| Covariate |  | Before propensity score matching | | | After propensity score matching | | |
| --- | --- | --- | --- | --- | --- | --- | --- |
|  |  | K^+^≤10mEq | K^+^>10mEq | SDiff | K^+^≤10mEq | K^+^>10mEq | SDiff |
|  |  | N = 94,598 | N = 84,838 |  | N = 94,593 | N = 84,832 |  |
| Age, in years | Overall |  |  | 0.02 |  |  | 0.02 |
|  | 18-44 | 14.4% | 14.1% | . | 14.4% | 14.1% | . |
|  | 45-54 | 13.8% | 14.4% | . | 13.8% | 14.5% | . |
|  | 55-64 | 14.5% | 15.6% | . | 14.5% | 15.6% | . |
|  | 65-74 | 20.6% | 20.7% | . | 20.6% | 20.7% | . |
|  | 75-84 | 22.6% | 21.9% | . | 22.6% | 21.9% | . |
|  | ≥85 | 14.1% | 13.2% | . | 14.1% | 13.3% | . |
| Sex | Female | 72.3% | 69.6% | 0.06 | 72.3% | 69.6% | 0.06 |
| Race | White | 52.7% | 56.6% | 0.08 | 52.7% | 56.6% | 0.08 |
|  | Black | 14.9% | 16.4% | 0.04 | 14.9% | 16.4% | 0.04 |
|  | Hispanic | 17.0% | 13.7% | 0.09 | 17.0% | 13.7% | 0.09 |
|  | Other/unknown | 15.5% | 13.2% | 0.06 | 15.5% | 13.2% | 0.06 |
| State of residence | CA | 42.1% | 35.1% | 0.14 | 42.1% | 35.1% | 0.14 |
|  | FL | 25.1% | 23.1% | 0.05 | 25.1% | 23.1% | 0.05 |
|  | NY | 13.4% | 15.6% | 0.06 | 13.5% | 15.6% | 0.06 |
|  | OH | 12.9% | 13.8% | 0.03 | 12.9% | 13.8% | 0.03 |
|  | PA | 6.5% | 12.3% | 0.20 | 6.5% | 12.3% | 0.20 |
| Year of cohort entry | 2000 | 12.6% | 14.8% | 0.06 | 12.6% | 14.8% | 0.06 |
|  | 2001 | 12.3% | 12.1% | <.01 | 12.3% | 12.1% | <.01 |
|  | 2002 | 13.1% | 13.4% | 0.01 | 13.1% | 13.4% | 0.01 |
|  | 2003 | 11.9% | 11.0% | 0.03 | 11.9% | 11.0% | 0.03 |
|  | 2004 | 11.2% | 10.4% | 0.03 | 11.2% | 10.4% | 0.03 |
|  | 2005 | 12.0% | 11.3% | 0.02 | 12.0% | 11.3% | 0.02 |
|  | 2006 | 15.6% | 16.4% | 0.02 | 15.6% | 16.4% | 0.02 |
|  | 2007 | 11.4% | 10.6% | 0.03 | 11.4% | 10.6% | 0.03 |
| Medicaid-Medicare dual eligibility status | Yes | 69.2% | 70.7% | 0.03 | 69.2% | 70.7% | 0.03 |
| Nursing home residence status | Yes | 14.7% | 16.0% | 0.04 | 14.7% | 16.0% | 0.04 |
| Cohort-initiating loop diuretic* | Bumetanide | 1.4% | 2.2% | . | 1.4% | 2.2% | . |
|  | Furosemide | 97.3% | 95.9% | . | 97.3% | 95.9% | . |
|  | Starting dose <40mg/d* | 56.6% | 24.1% | . | 56.6% | 24.1% | . |
|  | Starting dose =40mg/d* | 39.9% | 55.5% | . | 39.9% | 55.5% | . |
|  | Starting dose >40mg/d* | 3.5% | 20.4% | . | 3.5% | 20.4% | . |
|  | Torsemide | 1.3% | 1.9% | . | 1.3% | 1.9% | . |
| Lab monitoring of K+ in first 30 days of loop prescription* | Yes | 35.5% | 36.4% | . | 34.0% | 35.3% | . |
| Acidosis | Yes | 1.8% | 2.4% | 0.05 | 1.8% | 2.4% | 0.05 |
| Alcohol abuse** | Yes | 5.9% | 6.7% | 0.03 | 5.9% | 6.7% | 0.03 |
| Amyloidosis | Yes | 0.1% | 0.1% | <.01 | 0.1% | 0.1% | <.01 |
| Anemia | Yes | 43.2% | 43.2% | <.01 | 43.2% | 43.2% | <.01 |
| Arrhythmia/conduction disorder | Yes | 33.8% | 38.3% | 0.09 | 33.8% | 38.3% | 0.09 |
| Ascites | Yes | 1.4% | 1.9% | 0.05 | 1.4% | 1.9% | 0.05 |
| Asthma/COPD | Yes | 43.8% | 46.3% | 0.05 | 43.8% | 46.3% | 0.05 |
| Coronary artery disease | Yes | 46.5% | 51.4% | 0.10 | 46.5% | 51.4% | 0.10 |
| Cerebrovascular disease | Yes | 30.5% | 31.1% | 0.01 | 30.5% | 31.1% | 0.01 |
| Corticoadrenal insufficiency | Yes | 0.8% | 1.0% | 0.02 | 0.8% | 1.0% | 0.02 |
| Cushing’s syndrome | Yes | 0.2% | 0.3% | 0.01 | 0.2% | 0.3% | 0.01 |
| Diabetes insipidus | Yes | 0.1% | 0.2% | <.01 | 0.1% | 0.2% | <.01 |
| Diabetes mellitus | Yes | 44.7% | 45.9% | 0.03 | 44.7% | 45.9% | 0.03 |
| Mg^2+^ metabolism disorder | Yes | 1.5% | 2.0% | 0.03 | 1.5% | 2.0% | 0.03 |
| Edema | Yes | 26.3% | 27.9% | 0.04 | 26.3% | 27.9% | 0.04 |
| Glaucoma | Yes | 14.8% | 13.9% | 0.03 | 14.8% | 13.9% | 0.03 |
| Heart failure/cardiomyopathy | Yes | 37.6% | 47.4% | 0.20 | 37.6% | 47.4% | 0.20 |
| HIV/AIDS | Yes | 1.7% | 1.9% | 0.01 | 1.7% | 1.9% | 0.01 |
| Hypertension | Yes | 74.7% | 75.9% | 0.03 | 74.7% | 75.9% | 0.03 |
| Hypercholesterolemia | Yes | 53.4% | 52.2% | 0.02 | 53.4% | 52.2% | 0.02 |
| Hyperkalemia | Yes | 3.6% | 4.4% | 0.04 | 3.6% | 4.4% | 0.04 |
| Hyperosmolality | Yes | 1.3% | 1.5% | 0.02 | 1.3% | 1.5% | 0.02 |
| Hypokalemia | Yes | 8.8% | 12.2% | 0.11 | 8.8% | 12.2% | 0.11 |
| Hypothyroidism | Yes | 25.7% | 24.5% | 0.03 | 25.7% | 24.5% | 0.03 |
| Hyperthyroidism | Yes | 5.8% | 5.2% | 0.03 | 5.8% | 5.2% | 0.03 |
| Kidney disease | Yes | 23.5% | 25.2% | 0.04 | 23.5% | 25.2% | 0.04 |
| Liver disease | Yes | 17.1% | 16.8% | <.01 | 17.1% | 16.8% | <.01 |
| Alkalosis, metabolic | Yes | 0.4% | 0.6% | 0.02 | 0.4% | 0.6% | 0.02 |
| Nocturia | Yes | 2.3% | 2.0% | 0.02 | 2.3% | 2.0% | 0.02 |
| Obesity** | Yes | 17.0% | 19.1% | 0.06 | 17.0% | 19.1% | 0.06 |
| Uropathy, obstructive | Yes | 0.1% | 0.1% | <.01 | 0.1% | 0.1% | <.01 |
| Pulmonary circulation | Yes | 6.4% | 9.3% | 0.11 | 6.4% | 9.3% | 0.11 |
| Pulmonary congestion and hypostasis / pulmonary edema | Yes | 9.8% | 13.4% | 0.11 | 9.8% | 13.4% | 0.11 |
| Pyloric stenosis | Yes | 0.2% | 0.2% | <.01 | 0.2% | 0.2% | <.01 |
| Rheumatoid arthritis and other inflammatory polyarthropathies | Yes | 28.9% | 26.6% | 0.05 | 28.9% | 26.6% | 0.05 |
| Systemic lupus erythematosus | Yes | 1.2% | 1.3% | <.01 | 1.2% | 1.3% | <.01 |
| Sickle cell disease | Yes | 0.2% | 0.2% | <.01 | 0.2% | 0.2% | <.01 |
| Smoking, tobacco** | Yes | 14.0% | 16.2% | 0.06 | 14.0% | 16.2% | 0.06 |
| Substance abuse** | Yes | 5.3% | 5.8% | 0.02 | 5.3% | 5.8% | 0.02 |
| Valvular heart disease | Yes | 26.5% | 30.7% | 0.09 | 26.5% | 30.7% | 0.09 |
| ACEIs /ATIIRBs | Yes | 44.9% | 41.8% | 0.06 | 44.9% | 41.8% | 0.06 |
| Adrenergic bronchodilators | Yes | 34.6% | 32.5% | 0.04 | 34.6% | 32.5% | 0.04 |
| Anorexiants/antiobesity agents | Yes | 0.4% | 0.3% | 0.01 | 0.4% | 0.3% | 0.01 |
| Antidiabetics | Yes | 27.9% | 27.1% | 0.02 | 27.9% | 27.1% | 0.02 |
| Antiarrhythmics, type I, except lidocaine and phenytoin | Yes | 0.7% | 0.8% | <.01 | 0.7% | 0.8% | <.01 |
| Antiarrhythmics, type III | Yes | 2.0% | 2.1% | <.01 | 2.0% | 2.1% | <.01 |
| Antiadrenergic agents | Yes | 6.8% | 6.4% | 0.01 | 6.8% | 6.4% | 0.01 |
| Antiglaucoma agents, ophthalmic | Yes | 7.6% | 6.7% | 0.04 | 7.6% | 6.7% | 0.04 |
| Antiglaucoma agents, oral | Yes | 0.5% | 0.5% | <.01 | 0.5% | 0.5% | <.01 |
| Antihyperlipidemics | Yes | 36.0% | 33.0% | 0.06 | 36.0% | 33.0% | 0.06 |
| Antiretrovirals | Yes | 0.7% | 0.9% | 0.02 | 0.7% | 0.9% | 0.02 |
| Beta blockers, systemic | Yes | 31.1% | 29.7% | 0.03 | 31.1% | 29.7% | 0.03 |
| Bisphosphonates | Yes | 12.1% | 9.8% | 0.07 | 12.1% | 9.8% | 0.07 |
| Calcium channel blockers, non-verapamil | Yes | 33.1% | 30.6% | 0.05 | 33.1% | 30.6% | 0.05 |
| Corticosteroids, inhaled | Yes | 16.0% | 15.5% | 0.01 | 16.0% | 15.5% | 0.01 |
| Corticosteroids, oral | Yes | 22.7% | 21.7% | 0.02 | 22.7% | 21.7% | 0.02 |
| Digoxin | Yes | 7.0% | 7.3% | <.01 | 7.0% | 7.3% | <.01 |
| Diuretics, thiazides* | Yes | 33.6% | 31.5% | . | 33.6% | 31.5% | . |
| Immunosuppressives | Yes | 0.4% | 0.5% | 0.01 | 0.4% | 0.5% | 0.01 |
| Nitrates | Yes | 20.2% | 19.7% | 0.01 | 20.2% | 19.7% | 0.01 |
| Thyroid hormones | Yes | 11.9% | 10.7% | 0.04 | 11.9% | 10.7% | 0.04 |
| Vasodilators, non-nitrates | Yes | 0.8% | 1.0% | 0.01 | 0.8% | 1.0% | 0.01 |
| Calcium channel blocker, verapamil | Yes | 5.1% | 4.7% | 0.02 | 5.1% | 4.7% | 0.02 |
| Warfarin | Yes | 7.8% | 8.6% | 0.03 | 7.8% | 8.6% | 0.03 |
| Xanthine derivatives | Yes | 4.9% | 4.9% | <.01 | 4.9% | 4.9% | <.01 |

* not included in the propensity score

** health-related behavior or state ascertained via diagnostic codes alone

PS: propensity score; K^+^: empiric potassium supplementation; SDiff: standardized difference; COPD: chronic obstructive pulmonary disease; HIV: human immunodeficiency virus; AIDS: acquired immunodeficiency syndrome; Mg^2+^: magnesium; ACEIs: angiotensin-converting enzyme inhibitors; ATIIRBs: angiotensin-II receptor blockers
